# Contribution of major FLM isoforms to temperature-dependent flowering in *Arabidopsis thaliana*

**DOI:** 10.1093/jxb/erx328

**Published:** 2017-09-25

**Authors:** Giovanna Capovilla, Efthymia Symeonidi, Rui Wu, Markus Schmid

**Affiliations:** 1 Max Planck Institute for Developmental Biology, Department of Molecular Biology, Spemannstr. 35, Tübingen, Germany; 2 Umeå Plant Science Centre, Department of Plant Physiology, Umeå University, Umeå, Sweden

**Keywords:** *Arabidopsis thaliana*, CRISPR/Cas9, FLOWERING LOCUS M (FLM), flowering time, splice isoforms, temperature-dependent alternative splicing

## Abstract

*FLOWERING LOCUS M (FLM*), a component of the thermosensory flowering time pathway in *Arabidopsis thaliana*, is regulated by temperature-dependent alternative splicing (AS). The main splicing variant, FLM-β, is a well-documented floral repressor that is down-regulated in response to increasing ambient growth temperature. Two hypotheses have been formulated to explain how flowering time is modulated by AS of *FLM*. In the first model a second splice variant, FLM-δ, acts as a dominant negative isoform that competes with FLM-β at elevated ambient temperatures, thereby indirectly promoting flowering. Alternatively, it has been suggested that the induction of flowering at elevated temperatures is caused only by reduced *FLM-β* expression. To better understand the role of the two FLM splice forms, we employed CRISPR/Cas9 technology to specifically delete the exons that characterize each splice variant. Lines that produced repressive *FLM-β* but were incapable of producing *FLM-δ* were late flowering. In contrast, *FLM-β* knockout lines that still produced *FLM-δ* flowered early, but not earlier than the *flm-3* loss of function mutant, as would be expected if FLM-δ had a dominant-negative effect on flowering. Our data support the role of *FLM-β* as a flower repressor and provide evidence that a contribution of *FLM-δ* to the regulation of flowering time in wild-type *A. thaliana* seems unlikely.

## Teaser

Targeted deletions of exons in *FLM*, generated through CRISPR/Cas9 technology, clarify how specific splice variants contribute to the regulation of temperature-dependent flowering time in *Arabidopsis thaliana*.

## Introduction

The correct timing of the transition from vegetative growth to flowering is critical to ensure reproductive success. Due to its importance, flowering time is regulated by an intricate genetic network that integrates both endogenous and environmental signals such as photoperiod, namely day length, and temperature (Srikanth and Schmid, 2011).

Two aspects regarding the regulation of flowering by temperature can be distinguished: the response to prolonged periods of cold, such as overwintering and vernalization, and the effects of ambient temperature. In *Arabidopsis thaliana* vernalization controls flowering through the MADS domain transcription factor FLOWERING LOCUS C (FLC), which is epigenetically silenced in response to non-freezing temperatures (Gendall *et al.*, 2001; Bastow *et al.*, 2004).

The vernalization pathway not only protects plants from damage by preventing flowering in unfavourable winter conditions (Michaels and Amasino, 1999), but also synchronizes flowering of individuals the following spring. The molecular mechanisms by which ambient temperature modulates flowering time are not as well understood. However, the importance of ambient temperature is highlighted by the finding that, in *A. thaliana*, a moderate temperature increase from 23°C to 27°C is sufficient to induce flowering under an otherwise non-inductive short day photoperiod (Balasubramanian *et al.*, 2006). One of the genes involved in the thermosensory pathway in *A. thaliana* is *FLOWERING LOCUS M* (*FLM*), also called *MADS AFFECTING FLOWERING 1* (*MAF1*), which encodes a MADS-box transcription factor related to FLC. Loss of function *flm* mutants flower earlier than wild-type in both inductive and non-inductive photoperiods, whereas constitutive expression of *FLM* causes late flowering, indicating that FLM normally acts to repress flowering (Scortecci *et al.*, 2001). Like the majority of mRNAs in eukaryotic cells, *FLM* nascent transcripts undergo splicing and through the selection of alternative splice sites different mature mRNAs are produced from the same encoding gene (Scortecci *et al.*, 2001; Syed *et al.*, 2012; Kornblihtt *et al.*, 2013; Staiger, 2015).

Four AS variants, *FLM-α*, *FLM-β*, *FLM-γ*, and *FLM-δ*, with alternative second/third and eighth/ninth cassette exons have originally been described in Wassilewskjia (Ws) accession (Scortecci *et al.*, 2001). However, only two of these splice variants, *FLM-β* and *FLM-δ*, which differ in the inclusion of the mutually exclusive second and third exon and encode potentially functional proteins, were found to be abundant in Col-0 (Posé *et al.*, 2013).

Interestingly, *FLM* is alternatively spliced in response to ambient temperature (Balasubramanian *et al.*, 2006). *FLM-β* is expressed at higher levels at low ambient temperatures and decreases strongly in abundance as temperature increases (Posé *et al.*, 2013; Lee *et al.*, 2013). In contrast, *FLM-δ* has been shown to either not respond to changes in temperature (Lutz *et al.*, 2015; Sureshkumar *et al.*, 2016; Lutz *et al.*, 2017) or to be induced by elevated temperatures (Posé *et al.*, 2013). Additional splice variants have been identified in Col-0, particularly at elevated ambient temperatures (Sureshkumar *et al.*, 2016). These are the consequences of different combinations of intron retention and/or exon skipping, of which almost all harbour premature stop codons (Sureshkumar *et al.*, 2016).

Two hypotheses have been proposed to explain how the AS of *FLM* might control flowering time in *A. thaliana*. Based on the early flowering phenotype observed in overexpression lines in both Col-0 and the *flm-3* loss of function mutant, as well as biochemical evidence from electrophoretic mobility shift assays, it has been suggested that *FLM-δ* might act as a dominant negative version of FLM. According to this model FLM-δ competes at elevated temperatures with FLM-β for interaction partners such as SHORT VEGETATIVE PHASE (SVP) and prevents the resulting protein complexes from binding to and repressing its target genes, thereby indirectly promoting the transition to flowering (Lee *et al.*, 2013; Posé *et al.*, 2013). An important aspect of this hypothesis is that AS can convert the floral repressor FLM into an indirect activator of flowering, allowing the ratio between the two *FLM* isoforms to fine-tune flowering time. Alternatively, increasing ambient temperature has been suggested to induce flowering by reducing the expression of *FLM-β*, while at the same time boosting *FLM-δ* and non-canonical isoforms that mostly contain premature stop codons and are subsequently degraded by nonsense-mediated decay (Lykke-Andersen and Jensen, 2015; Sureshkumar *et al.*, 2016). Both models have in common that the relative abundance of the floral repressor FLM-β decreases at elevated temperatures; the contribution of FLM-δ in the regulation of flowering is more controversial.

To determine the *in vivo* role of the two main isoforms of *FLM* in the Col-0 accession, we employed CRISPR/Cas9 to edit the endogenous *FLM* genomic sequence (Gasiunas *et al.*, 2012; Jinek *et al.*, 2012). The *FLM* deletion lines engineered lack either the second (FLM-ΔE2) or third (FLM-ΔE3) exon. They therefore express only one of the two major splice variants, *FLM-β* in FLM-ΔE3 and *FLM-δ* in FLM-ΔE2 plants. As expected, FLM-ΔE3 plants flowered later than wild-type plants, confirming the role of FLM-β as a floral repressor. In contrast, FLM-ΔE2 lines flowered early, but not earlier than *flm-3*, suggesting that under normal growth conditions FLM-δ does not exert a dominant negative function in Col-0. Temperature-dependent AS of *FLM* and its effect on flowering time appears to be conserved as we observed a decrease in the expression of *FLM-β* in response to increasing the ambient temperature and consequent temperature sensitive flowering time in 33 natural accessions of *A. thaliana*.

## Materials and methods

### Plant material and growth conditions

Seeds were surface sterilized with 20 mL of thin bleach and 600 μL of 37.5% HCl for 4 h followed by 1.5 h in a laminar flow to evaporate chlorine gas and then stratification in 0.1% agar at 4°C in the dark for 72 h before being planted directly on soil. Seeds from natural accessions, listed in Supplementary Table S1 at *JXB* online, belong to the 1001 genomes project, and were obtained from colleagues at the MPI for Developmental Biology, Tuebingen, Germany. The following lines have been previously published (Posé *et al.*, 2013): *flm-3, flm-3* 35S:*FLM-δ* #3, and *flm-3* 35S:*FLM-δ* #43. Plants were grown in soil in long day conditions (LD), namely 16 h light/8 h dark, at a specified temperature either in Percival chambers or in growth rooms. To analyze variation in splicing patterns in response to a change in temperature, plants were grown for 9 d at 23°C in LD and then shifted to 16°C, 27°C or kept at 23°C in LD for 3 d. Three pools of 10 seedlings for each line in each temperature were collected after the shift at zeitgeber time 6 and snap frozen in liquid nitrogen. To analyze flowering time, plants were grown at 16°C, 23°C, or 27°C and the days to flower as well as the rosette and cauline leaf number were recorded.

### RNA extraction and cDNA synthesis

RNA was extracted with TRIzol® reagent or 5:1 acidic phenol:chloroform as previously described (Box *et al.*, 2011). RNA concentration and purity were determined by using a Nanodrop ND-2000 spectrophotometer (Nanodrop Technologies) and only high quality RNA samples, with A260/A230>2.0 and A260/A280>1.8, were used for subsequent experiments. To remove possible DNA contamination, RNA samples were treated with DNaseI (Thermo Scientific) for 30 min at 37°C. 3 μg RNA was used for complementary DNA (cDNA) synthesis using the RevertAid First Strand cDNA Synthesis kit in accordance with the manufacturer’s instructions (Thermo Scientific).

### TaqMan assay and SYBR green qPCR

Multicolour TaqMan analysis was carried out in 384-well plates to measure the relative expression of *FLM* splice variants that contained either the second or third exon. TaqMan technology exploits the 5’ nuclease activity of Taq DNA polymerase (Holland *et al.*, 1991) and probes designed on the transcript of interest were labelled with fluorescent reporters with non-overlapping detection spectra at the 5’ end and a quencher dye at the 3’ end. Primers and probes are listed in Supplementary Table S2, which also includes details about quenchers and detection channels. iQ^TM^ Multiplex Powermix (BioRad) was used for the PCR following the manufacturer’s instructions and carried out using an annealing temperature of 58°C. Real time PCR using SYBR green chemistry (Thermo Scientific) were run as previously described for TaqMan, the primer sequences are listed in Supplementary Table S3. The exponential amplification of the fluorescence intensity was measured and quantified (Lee *et al.*, 1993) using a BioRad C1000 Touch Thermal Cycler. The relative expressions have been calculated with the delta Ct method and performed in technical triplicates for each of the three biological replicates.

### Plasmid construction and plant transformation

The two CRISPR/Cas9 vectors used in this study, pGC001 and pGC002, were assembled using the GreenGate system (Lampropoulos *et al.*, 2013). The final constructs contain p*EC1.1*::AthCas9:trbcs assembled from GreenGate modules A: *A. thaliana* pEC1.1; B: A. *thaliana* codon-optimized Cas9; and C: rbcs terminator, the sgRNAs listed in Supplementary Table S3 under the control of the *A. thaliana U6* promoter (GreenGate modules D and E), and a pAt2S3::mCherry:tMAS cassette (GreenGate module F) for seed selection as described by Gao and colleagues (2016). The p*35S*::g*FLM* vector (GC003) was also assembled using the GreenGate system. For this, the genomic region of *FLM*, including untranslated regions (UTRs), was amplified from Col-0 seedlings and cloned into the GreenGate module C entry vector (Lampropoulos *et al.*, 2013). The final GreenGate reaction was performed using modules A: p35S, B: empty (pGGB003), the C module carrying the full genomic *FLM*, D: empty (pGGD002), E: rbcs terminator, and BASTA resistance as a selection marker (module F; pGGF001). pGGZ001 was used as destination vector for all the GreenGate reactions described. Plants grown at 23°C were transformed by floral dipping using *Agrobacterium tumefaciens*-mediated gene transfer according to standard protocols (Clough and Bent, 1998). Transformants were selected by fluorescence microscopic identification of mCherry-positive seeds as previously reported in Gao *et al.* (2016) or by BASTA selection.

### Sanger sequencing of *FLM* clones

The *FLM* open reading frame was amplified using primers 5′ CGCTGTTGTCGTCGTATCTG 3′ and 5′ CAGCAACGTATTCTTTCCCAT 3′ from the same cDNA used for the TaqMan assay described above. The PCR products were subsequently cloned into pGem^®^-T Vector System I according to the manufacturer’s instructions (Promega) and individual clones were sequenced using Sanger sequencing.

## Results

### Deletion of isoform-specific *FLM* exons by CRISPR/Cas9

**Fig. 1.**
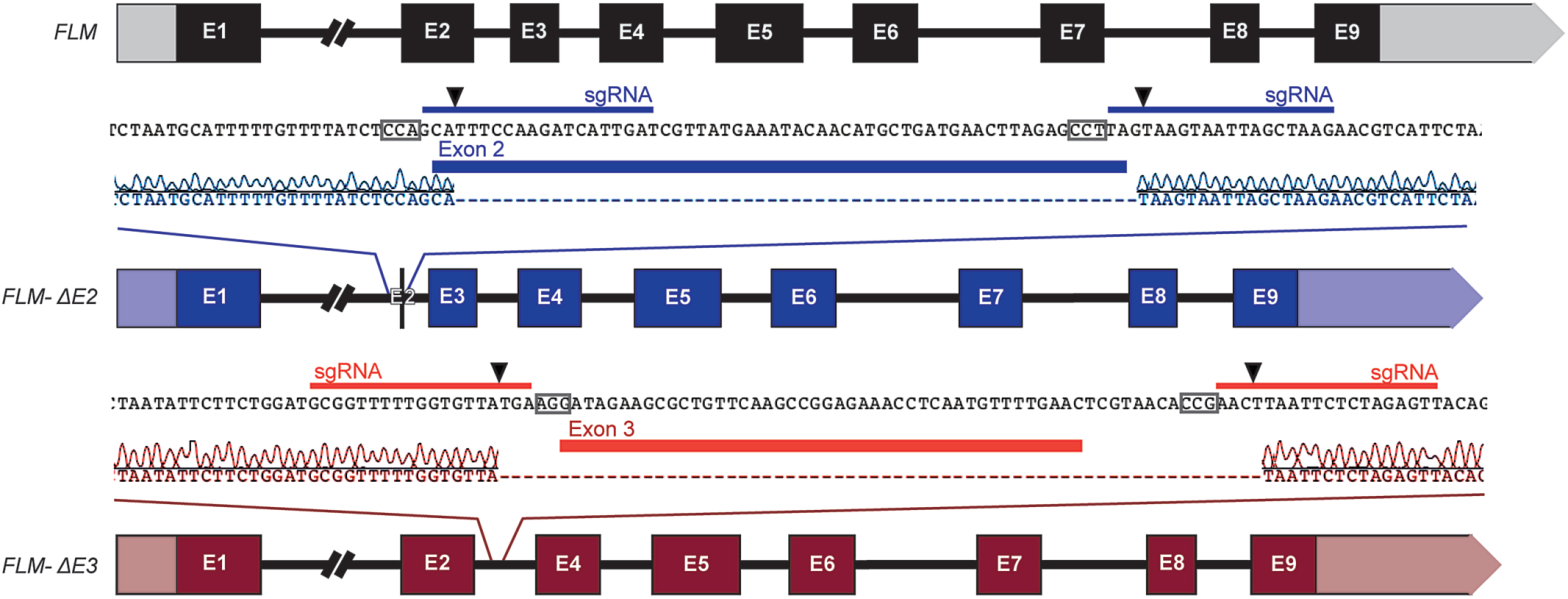
Schematic representation of the *FLM* locus in the two *FLM* CRISPR lines. The annotated *FLM* gene structure is shown in black, exons are marked as squares and introns as straight lines. The edited FLM-ΔE2 and FLM-ΔE3 lines are represented in blue and red, respectively. Close-ups provide detailed information on the position of the deletions as determined by Sanger sequencing of the CRISPR lines aligned with wild-type *FLM* genomic sequences. Position of exons 2 and 3 are marked by thick blue and red lines, respectively. sgRNAs are represented by thin blue and red lines, PAM sites are marked by grey boxes on the wild-type sequences and the position of the expected Cas9 cuts are indicated with black triangles.

Targeted mutations were obtained in the *FLM* genomic sequence in the Col-0 background using CRISPR/Cas9. We employed the *A. thaliana EGG CELL1* (*EC1.1*) promoter to express an Arabidopsis codon optimized Cas9 (Fauser *et al.*, 2014) in the egg cell as previously described (Wang *et al.*, 2015) and the *U6* promoter (Waibel and Filipowicz, 1990) to express sgRNAs corresponding to regions flanking the second or third exon, respectively (Fig. 1). FLM-ΔE2 carries a deletion of 57 bp that covers most of exon 2, which is normally incorporated in the repressive *FLM-β* splice variant, apart from 2 bp at the 5’ end and the first 2 bp of intron 3 (Fig. 1). FLM-ΔE3 carries a 64 bp deletion that completely covers exon 3, normally found in *FLM-δ*, and short regions on the flanking introns (Fig. 1). *Cas9*-free lines homozygous for the deletion were recovered in the T3 generation using *mCherry*-based selection as previously reported (Gao *et al.*, 2016) (Supplementary Fig. S1). In the FLM-ΔE2 line the deletion is positioned exactly where predicted, with the Cas9 cuts located three bases distance from the protospacer adjacent motifs (PAMs) of the two sgRNAs (Fig. 1). Similarly, the deletion of the third exon in FLM-ΔE3 starts exactly where predicted but an additional thymine is deleted at the 3’ end of the sgRNA, at the transition between exon 3 and intron 3 (Fig. 1). Importantly, both edited lines did not carry any deletions or point mutations in the four genes most closely related to *FLM*, *MAF2* through *MAF*5, as verified by Sanger sequencing (data not shown).

### Diversity of *FLM* splice variants in Col-0 and CRISPR lines

**Fig. 2.**
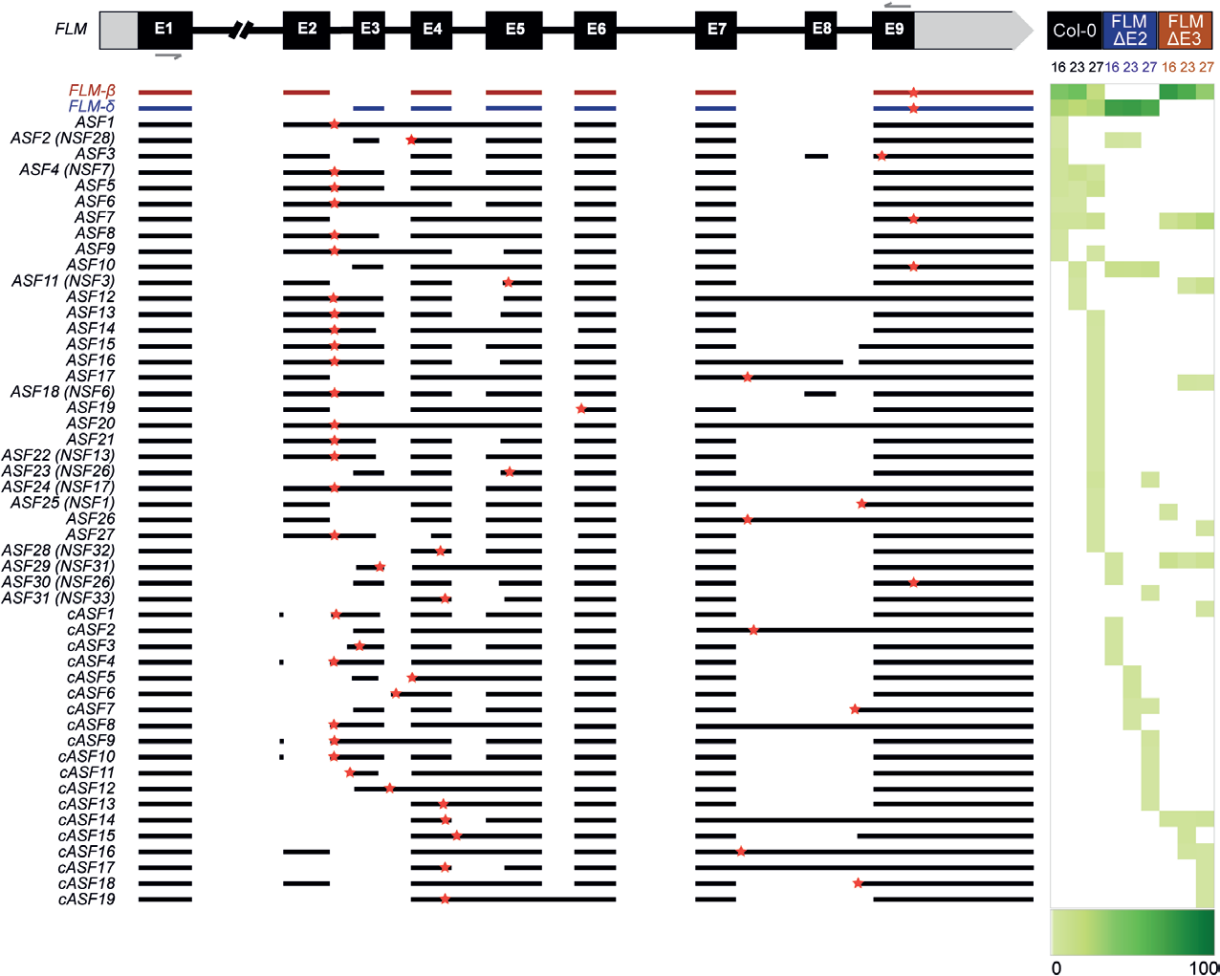
Alternative *FLM* splice variants detected by Sanger sequencing. The sequences present in the isoforms are aligned to the annotated *FLM* gene and grey arrows show the positions of the primers used to amplify the cDNA. Stop codons are represented as red stars. Isoforms identified in both Col-0 and at least in one of the CRISPR lines are listed as alternative splice forms (ASF) 1 to 31. The identifiers of isoforms previously described by Sureshkumar and colleagues (Sureshkumar *et al.*, 2016) are given in brackets. 19 new splice variants detected only in the CRISPR lines are listed as cASF. The heat map shows the frequency of each isoform in Col-0, FLM-ΔE2, and FLM-ΔE3 at 16°C, 23°C, and 27°C. The heat map legend shows a gradient of white to green where 0% of the sequences analysed is white and 100% is dark green.

To investigate the effect of the Cas9-induced deletions on *FLM* pre-mRNA splicing we amplified the *FLM* coding sequence by RT-PCR from Col-0 (control), FLM-ΔE2, and FLM-ΔE3 plants grown at 16°C, 23°C, and 27°C. The PCR products were cloned and 34 to 58 colonies for each line and each temperature were analyzed by Sanger sequencing. In agreement with previous results, the most abundant isoforms identified in Col-0 were *FLM-β* or *FLM-δ*, depending on the temperature, with *FLM-β* dominating at low temperatures and *FLM-δ* prevailing at 27°C (Fig. 2 and Supplementary Table S4) (Posé *et al.*, 2013). In addition, the frequency of non-canonical isoforms also increased in Col-0 from 30% at 16°C to over 50% at 27°C (Supplementary Table S4) as previously reported (Sureshkumar *et al.*, 2016). In FLM-ΔE2 we never detected *FLM-β* and *FLM-δ* was the most abundant isoform at 66–70% at all three temperatures (Fig. 2 and Supplementary Table S4). Non-canonical splicing variants increased from 29% to 34% in total, but each individual splice form accounted for only 2% to 12% of all cloned transcripts (Fig. 2 and Supplementary Table S4). In contrast, *FLM-β* was the predominant isoform in FLM-ΔE3 at each of the three temperatures, whereas *FLM-δ* could not be detected. The frequency of *FLM-β* decreased from 74% at 16°C to 35% at 27°C, while other isoforms increased from 25% to 64%. At elevated temperatures, the second most abundant isoform detected by sequencing was *ASF7*, which accounted for 25% at 27°C and 15% at 23°C. *ASF*7 differs from *FLM-β* only by the retention of the fourth intron, which does not cause any shift in the coding frame. Similarly, *ASF10* differs from *FLM-δ* by the very same in frame intron retention. In contrast, the other non-canonical isoforms accounted for only 2% and 10% of all splice variants detected in the FLM-ΔE3 line (Fig. 2 and Supplementary Table S4). In total, 31 alternatively spliced forms of *FLM* were found in Col-0 and at least one of the CRISPR lines, while 19 isoforms, *cASF1–19*, were detected exclusively in the CRISPR lines (Fig. 2). It should be noted, however, that we did not detect all splice variants that have been previously reported (Sureshkumar *et al.*, 2016), indicating that our sequencing approach does not cover the entire complexity of temperature-dependent splicing of *FLM*. Nevertheless, these results confirm that *FLM-β* and *FLM-δ* are the most abundant single *FLM* isoforms. In addition, our findings demonstrate that targeted deletion of either the second or third exon of *FLM* by CRIPSR/Cas9 results in plants that predominantly produce either *FLM-β* (FLM-ΔE3) or *FLM-δ* (FLM-ΔE2).

### Quantification of major *FLM* isoforms

**Fig. 3.**
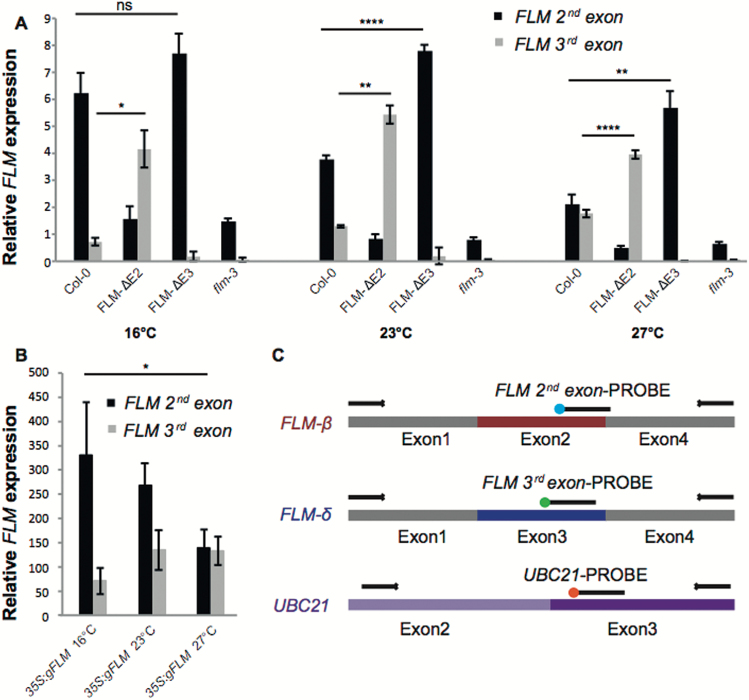
Relative expression of *FLM* main isoforms. A) Relative expression of transcripts containing the second (e.g. *FLM-β*) or third (e.g. *FLM-δ*) exon measured using the TaqMan assay in Col-0, the CRISPR lines, and the *flm-3* mutant at 16°C, 23°C, and 27°C. *P<0.05; **P<0.01;***P<0.001; ****P<0.0001; ns, not significant, using Anova and TukeyHSD test. B) Quantification of splice variants with the second exon and third exon of *FLM* by the TaqMan assay in a genomic *35S:gFLM* overexpression line at 16°C, 23°C, and 27 °C. For each sample three biological replicates of 10 seedlings each were used and each measurement has been replicated three times. Error bars designate the standard deviation between biological replicates. C) Schematic representation of the TaqMan assay designed to detect splice variants with the second exon and third exon of *FLM* (e.g. *FLM-β* and *FLM-δ*) and the normalization control *UBC21*. Primers are shown as black arrows, probes as black segments. Fluorophores are marked as blue (6-FAM™), green (HEX™2), and orange (CY5^®^).

To facilitate quantification of *FLM* isoforms we established a multicolour TaqMan assay to measure the relative expression of transcripts containing either the second or third exon, such as *FLM-β* and *FLM-δ*. Primers were designed in conserved regions within exon 1 and exon 4 of *FLM* and probes labelled with fluorophores with non-overlapping detection ranges, 6-FAM™ and HEX™2, were placed on exons 2 and 3, respectively (Fig. 3C). Expression of *UBC2*1 was used for normalization and was detected using a probe labelled with CY5^®^ whose emission spectra is not overlapping with the other two fluorophores used in this assay (Fig. 3C and Supplementary Table S2).

Using this multicolour TaqMan assay we found that the abundance of transcripts containing the second exon, such as *FLM-β*, decreased in Col-0 in response to increasing temperature (Fig. 3A). In contrast, transcripts containing the third exon, such as *FLM-δ*, showed a moderate increase (Fig. 3A). Despite the overall higher expression, temperature-dependent AS of *FLM* was also observed in a transgenic line that expresses the genomic region of *FLM*, including UTRs, under the control of the constitutive *35S* promoter (Fig. 3B). These findings confirm previous results (Posé *et al.*, 2013) and demonstrate the functionality of the TaqMan assay. However, we observed that the TaqMan probe against the second exon of *FLM* generates a weak signal even in the *flm-3* loss of function allele and in the FLM-ΔE2 line (Fig. 3A). Sanger sequencing of the PCR product confirmed that this signal was due to amplification of a fragment of *MAF2*.

Interestingly, the FLM-ΔE2 line displayed higher levels of exon 3-containing transcripts than Col-0 at all temperatures (Fig. 3A). This trend was even more pronounced in the FLM-ΔE3 plants, which showed elevated exon 2 levels (Fig. 3A). These findings suggest that deletion of either exon 2 or exon 3 does not negatively affect the basal expression of *FLM* but rather affects the relative ratio of the splice variants.

### Quantification of the non-canonical *FLM* isoforms *ASF7* and *ASF10*

**Fig. 4.**
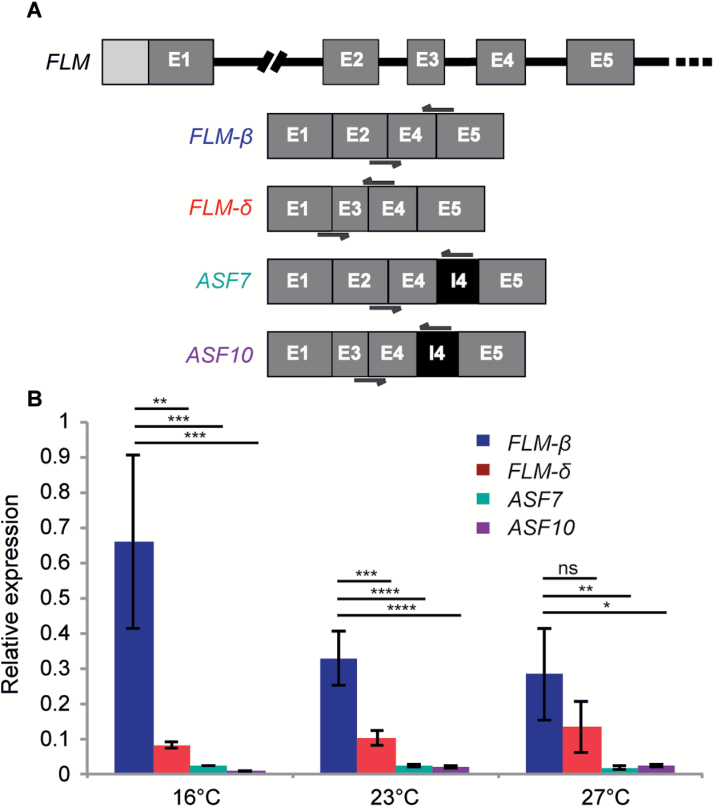
Relative expression of potential protein coding *FLM* isoforms. A) Representation of the annotated portion of *FLM* gene, from exon 1 to exon 6, and of the introns or exons included in the isoforms *FLM-β*, *FLM-δ*, *ASF7*, and *ASF10*. The primers used for the quantification of each isoform are represented as black arrows. B) Relative expression of each isoform at 16°C, 23°C, and 27°C. Error bars designate the standard deviation between three biological replicates. *P<0.05; **P<0.01; ***P<0.001; ****P<0.0001; ns, not significant, using Anova and TukeyHSD test.

We next quantified the relative expression of the two additional potentially protein-coding non-canonical isoforms, *ASF7* and *ASF10*, to evaluate their potential for contributing to the regulation of flowering. For this purpose we designed primers as specifically as possible (Fig. 4A) and performed RT PCR using SYBR technology. *UBC21* was used for normalization purposes and *FLM-β* and *FLM-δ* were included for comparison with the results obtained from the TaqMan assay. In agreement with our previous results (Fig. 3A) we observed decreased expression of *FLM-β* in response to elevated ambient temperature, whereas *FLM-δ* levels increased slightly. Importantly, the relative expression of the two non-canonical isoforms, *ASF7* and *ASF10*, was very low in comparison with *FLM-β* (Fig. 4B), suggesting that these isoforms play only a minor role, if any, in the regulation of flowering in Col-0.

### Flowering time of *35S:gFLM* and *FLM* CRISPR lines

**Fig. 5.**
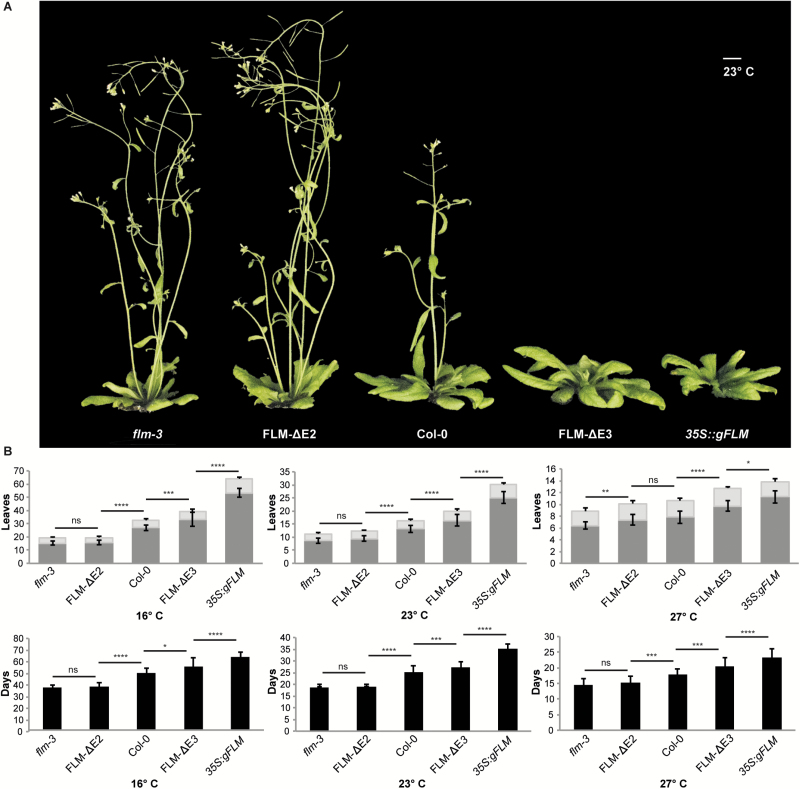
Flowering time of *FLM* CRISPR/Cas9 lines. A) Representative pictures of *flm-3*, FLM-ΔE2, Col-0, FLM-ΔE3, and *35S::gFLM* lines grown at 23°C in LD. Scale bar, 1 cm. B) Flowering time given as number of leaves (dark grey, rosette leaves; light grey, cauline leaves) and days to flowering (black) of plants grown at 16°C, 23°C, and 27°C. Error bars indicate standard deviation. *P<0.05; **P<0.01; ***P<0.001; ****P<0.0001; ns, not significant, using Anova and TukeyHSD test.

Next we determined the flowering time of the *35S:gFLM* and the *FLM* CRISPR lines at 16°C, 23°C, and 27°C. Similar to previous results (Scortecci *et al.*, 2001), constitutive expression of *gFLM* delayed flowering in all conditions tested, but the effect was more pronounced at 16°C than at 23°C or 27°C (Fig. 5, Supplementary Fig. S2, Supplementary Tables S5 and S7), possibly due to the reduction in exon 2-containing transcripts relative to exon 3-containing transcripts at 27°C (Fig. 3B). Confirming the importance of exon 2 for the repressive function of FLM, FLM-ΔE3 lines, which display higher expression levels of exon 2-containing transcripts (Fig. 3A), were also late flowering when compared with Col-0, but not as late as the *35S:gFLM* plants (Fig. 5 and Supplementary Table S5). In contrast, FLM-ΔE2 plants, which produce relatively high levels of *FLM-δ* (Fig. 3A), flowered earlier than Col-0, particularly at 16°C and 23°C, but not earlier than the *flm-3* mutant as one would have expected if *FLM-δ* had a dominant negative effect on flowering time (Fig. 5 and Supplementary Table S5).

### Constitutive expression of *FLM-δ* promotes flowering in *flm-3*

**Fig. 6.**
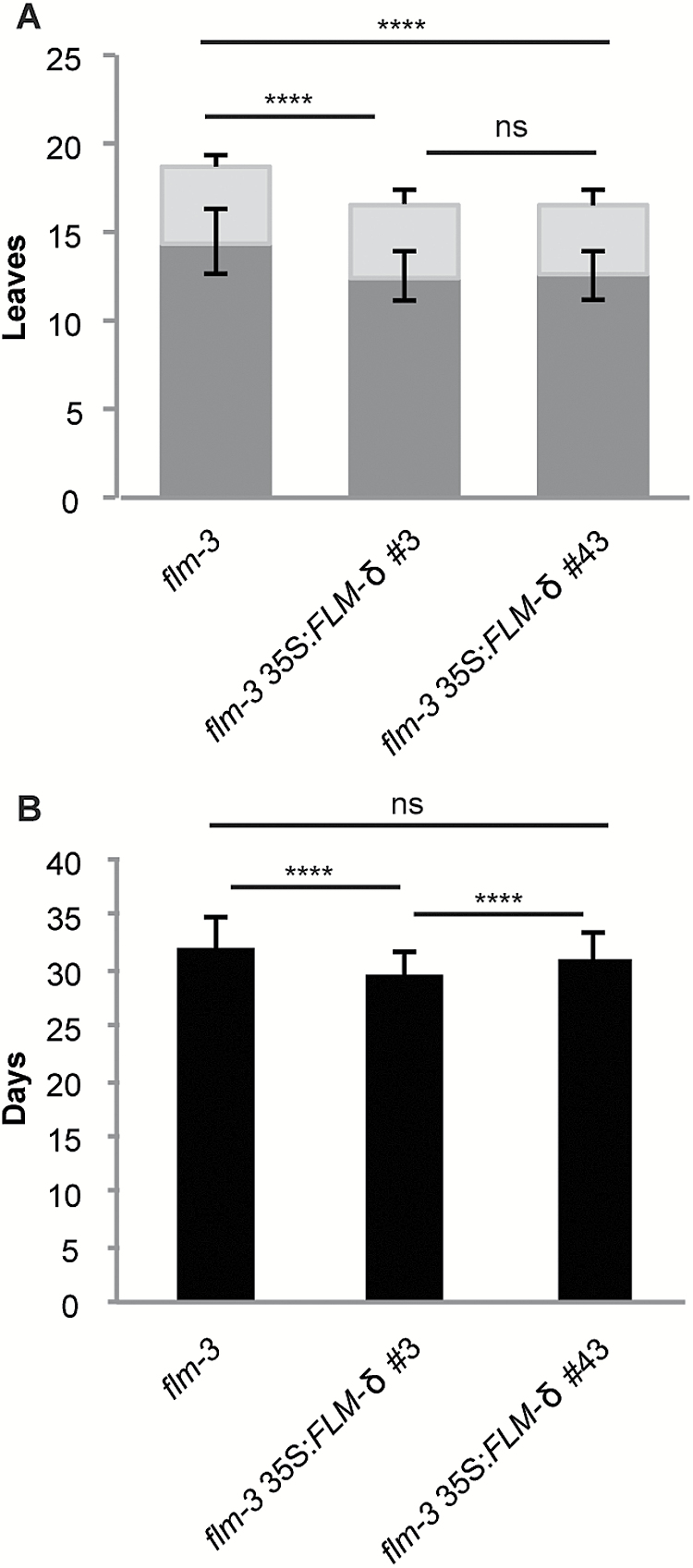
Flowering time of *FLM-δ* overexpression lines at 16°C. A) Flowering time given as number of leaves (dark grey, rosette leaves; light grey, cauline leaves). B) Days to flowering (black) of plants grown at 16°C. Error bars indicate standard deviation. *P<0.05; **P<0.01; ***P<0.001; ****P<0.0001; ns, not significant, using Anova and TukeyHSD test.

The data presented above indicate that the reduction in *FLM-β* rather than a dominant negative effect of *FLM-δ* causes plants to flower earlier at elevated ambient temperatures. However, when grown in LD at 16°C to maximize the differences in flowering time, plants expressing the *FLM-δ* open reading frame under the constitutive *35S* promoter in the *flm-3* background flowered significantly earlier than control plants (Fig. 6), confirming previously published results (Posé *et al.*, 2013). Taken together, these findings suggest that *FLM-δ* can have a dominant negative effect on flowering time under controlled growth conditions when expressed at high levels. However, it remains to be determined whether *FLM-δ* can contribute to the regulation of flowering in more natural settings, in other accessions, or under different growth conditions.

### *FLM* splicing in *A. thaliana* accessions

**Fig. 7.**
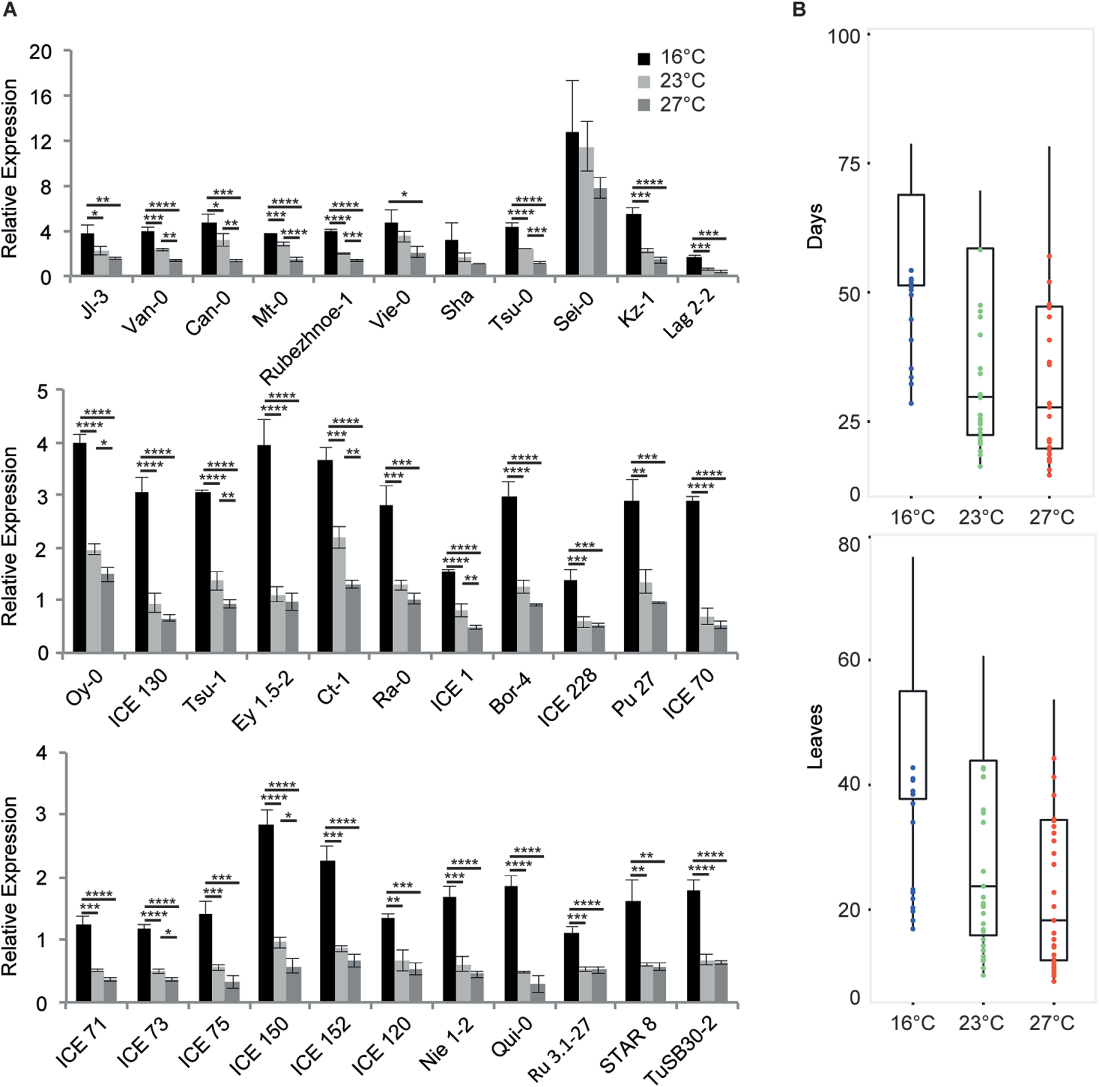
Expression of transcripts containing the second exon in natural accessions and flowering time. A) Relative expression of transcripts containing the second exon, e.g. *FLM-β*, measured using the TaqMan assay in 33 natural *A. thaliana* accessions. Error bars indicate standard deviation. *P<0.05; **P<0.01; ***P<0.001; ****P<0.0001, using Anova and TukeyHSD test. B) Mean flowering time in days and leaf numbers of 33 accessions showing Col-0-like temperature-dependent *FLM* splicing.

To investigate the conserved role of *FLM* splicing in natural strains we performed the TaqMan assays in 33 non-vernalization-requiring *A. thaliana* accessions grown at 16°C, 23°C, and 27°C (Supplementary Table S1). Expression of *FLM* splice variants containing the second exon, such as *FLM-β*, showed a trend similar to that observed in Col-0 in that it decreased at higher temperatures in all the 33 accessions analyzed (Fig 7A). The flowering time behaviour of these natural accessions was also largely consistent and comparable to Col-0: plants flowered later at low temperatures and earlier at high temperatures, when both the total leaf number and days to flowering are considered (Fig. 7B). Taken together these results suggest a conserved role for *FLM-β* in the regulation of flowering time, which is in line with a recent report that suggested that certain polymorphisms in *FLM* predict flowering in natural accessions of *A. thaliana* (Lutz *et al.*, 2017).

## Discussion

Over the past few years, substantial progress has been made in understanding the molecular mechanisms that control flowering in response to changes in ambient temperature and the role the MADS-domain transcription factor FLM plays in this process. At first identified as a floral repressor (Scortecci *et al.*, 2001), *FLM* has since been associated with the thermal induction of flowering time and the *FLM* transcript has been shown to be subject to temperature dependent alternative splicing (Balasubramanian *et al.*, 2006). Subsequently, one of these splice variants, FLM-β, was identified as the isoform that in complex with SVP represses floral transition by directly binding to regulatory elements important in flowering time and floral homeotic genes (Posé *et al.*, 2013; Lee *et al.*, 2013). How temperature-dependent alternative splicing contributes to the induction of flowering particularly at higher temperatures is, however, controversial. One model suggested that elevated ambient temperatures promote the expression of a second isoform, FLM-δ, that competes with FLM-β for interaction with SVP, resulting in the formation of a non-functional FLM-δ/SVP complex, thereby indirectly inducing flowering (Posé *et al.*, 2013). However, evidence for the potential dominant-negative effect of FLM-δ on flowering time largely stems from strong overexpression of this isoform from the constitutive *35S* promoter (Posé *et al.*, 2013), which is a highly artificial context. Indeed, the importance of FLM-δ in regulating flowering has recently been challenged. Instead it has been proposed that elevated temperature induces the production of non-canonical *FLM* isoforms that are targeted for degradation by nonsense mediated-decay, thereby decreasing the level of the functional isoform, FLM-β (Sureshkumar *et al.*, 2016).

To establish to what extent the two most abundant *FLM* variants contribute to the thermosensitive regulation of flowering in a system as close as possible to wild-type, we employed CRISPR/Cas9 to edit the endogenous *FLM* locus by creating targeted deletions of the second or third exon. The resulting Cas9-free lines, FLM-ΔE2 and FLM-ΔE3 (Fig. 1), did not express *FLM-β* and *FLM-δ*, respectively, and the expression of other isoforms was not noticeably compromised (Fig. 2). Nevertheless, it could be argued that the CRISPR lines do not represent a completely unbiased solution since we detected 19 new isoforms that have not been previously reported in Col-0 (Fig. 2), probably as a consequence of the changes made to the *FLM* genomic sequence. Alternatively, the detection of new isoforms could also indicate that the complexity of *FLM* splice variants is not fully captured by the low-throughput Sanger sequencing approach used in our study and by Sureshkumar and colleagues (Sureshkumar *et al.*, 2016). However, all the new isoforms were only detectable at a low frequency (Supplementary Table S4) and, similar to the majority of those shared with the wild-type, contained premature termination codons. Importantly, the expression of *FLM-β* was still down-regulated in the FLM-ΔE3 line in response to increasing temperature (Fig. 3A and Supplementary Table S4). Moreover, and in agreement with a previous report (Sureshkumar *et al.*, 2016), we observed an increase in the frequency of non-canonical isoforms at elevated temperatures (Supplementary Table S4) in both the wild-type and the edited lines. Taken together these findings strongly suggest that deleting the second or third exon had no major effect on overall *FLM* expression but rather shunted RNA molecules to other isoforms (Fig. 3A).

Probably as a consequence of the inability of FLM-ΔE3 to produce third exon-containing transcripts, the line showed elevated expression of transcripts containing the second exon (*FLM-β*) (Fig. 3A). This is in agreement with a hypothesis first formulated by Sureshkumar and colleagues (Sureshkumar *et al.*, 2016) and results in a significant delay of the edited line in flowering when compared with wild-type, yet not as extreme as the transgenic lines overexpressing *gFLM*, especially at low ambient temperatures (Fig. 5). In the *35S:gFLM* line, constitutive expression of *gFLM* (Fig. 3B) delayed flowering in all conditions tested, which is in agreement with a previous report (Scortecci *et al.*, 2001), but the effect was less pronounced at 27°C, possibly due to the concomitant increase of exon 3-containing transcripts (Fig. 3B) that might buffer the overexpression of the functional repressor. Together these results demonstrate that the CRISPR lines can be employed to address specific questions regarding the contribution of *FLM* splicing variants in the regulation of flowering time.

The CRISPR lines enabled us to re-evaluate the contribution of FLM-*δ*, which is expressed at higher levels in FLM-ΔE2 than in wild-type in all conditions tested, in the regulation of flowering (Fig. 3A). As expected, these plants always flowered earlier than wild-type, which can easily be explained by their inability to produce the floral repressor FLM-β, but, surprisingly, never earlier than *flm-3* (Fig. 5). In contrast, early flowering was observed in two independent *FLM-δ* overexpression lines as originally described (Posé *et al.*, 2013). Overall these results indicate that *FLM-δ* in principle has the potential to act as a dominant regulator of flowering time when expressed at non-physiologically high levels. However, in Col-0 under the conditions tested, *FLM-δ* apparently never reaches the expression levels required to realize this potential.

If reducing levels of *FLM-β* were sufficient to promote flowering, the question arises of why plants invest in producing a plethora of alternatively spliced transcripts rather than just shutting down *FLM* expression altogether? It could simply be that evolution works with what is available and that in this case, maybe because of the structure of the *FLM* transcript, evolving temperature-dependent alternative splicing was the easiest solution to the problem. Alternatively, maintaining the ability to produce a variety of *FLM* isoforms could provide flowering time plasticity, in fact it seems possible that some of the alternative transcripts produced might play an active role in the regulation of flowering. In some conditions the FLM-ΔE2 lines actually flowered moderately late when compared with *flm-3* (Fig. 5) especially at elevated temperatures, even though no *FLM-β* expression could be detected (Fig. 2), suggesting that some *FLM* isoforms lacking exon 2 might contribute to the repression of flowering. Among the non-canonical isoforms detected, *ASF7* and *ASF10* clearly stand out as they have been detected relatively frequently at various temperatures especially in the CRISPRs lines. Furthermore, the structures of *ASF7* and *ASF10* differ from *FLM-β* and *FLM-δ*, respectively, only by the retention of intron 4, which does not result in a frame shift. *ASF7* and *ASF10* could therefore give rise to potentially functional proteins (Fig. 2). However, the expression levels of these two isoforms in Col-0 were extremely low (Fig. 4), making it seem unlikely that *ASF7* and/or *ASF10* play a major role in the regulation of flowering time. Together these findings suggest a potential role for *FLM* in contributing to the plasticity of flowering, which could be of relevance from an evolutionary perspective. In this context it is interesting to note that it was recently shown that natural haplotypes of *FLM* fine-tune flowering in Arabidopsis accessions (Lutz *et al.*, 2017).

Furthermore, it is noteworthy that the temperature-dependent AS of *FLM*, which leads to the down-regulation of *FLM-β*, seems to be well conserved in many natural rapid cycling accessions, characterised by non-functional *FRIGIDA (FRI*) and/or *FLC* alleles (Fig. 7A). In fact, *FLM-β* expression decreased in warmer temperatures in all the 33 natural accessions tested; this behaviour also correlated with flowering time and, like Col-0, the natural accessions showed accelerated flowering in response to elevated ambient temperatures (Fig. 7B and Supplementary Table S6).

In summary, it can be concluded that temperature-dependent AS splicing of *FLM* and its contribution to flowering in Col-0 and in many natural accessions can be largely explained by the role of *FLM-β* alone, which clearly represents the main flower-repressive isoform. However, why plants produce a large variation of non-canonical isoforms, particularly at elevated ambient temperatures, remains puzzling. It seems possible that, even though none of the individual splice variants reach a sufficient level of expression to affect flowering, in combination they might aid the plant in adapting to fluctuating temperature conditions.

## Supporting Information

Supplementary data are available at *JXB* online.

Fig. S1. Detection of CRISPR/Cas9-induced deletions in *FLM*.

Fig. S2. Flowering time of *FLM* transgenic lines and mutants at 16 °C.

Table S1. List of natural accessions analysed.

Table S2. Oligonucleotides used for TaqMan assay.

Table S3. Oligonucleotides used in this work.

Table S4. Percentage of *FLM* isoforms analysed in Col-0 and CRISPR lines at 16 °C, 23 °C and 27 °C.

Table S5. Flowering time of CRISPR lines.

Table S6. Flowering time of natural accessions.

Table S7. Flowering time data of FLM transgenic lines and mutants at 16 °C long days.

## Supplementary Material

Supplementary_Figures_S1_S2_Supplementary_Tables_S1_S7

## Abbreviations:

AS: alternative splicing
FLC: FLOWERING LOCUS C
FLM: FLOWERING LOCUS M
LD: long day conditions
SVP: SHORT VEGETATIVE PHASE
