# Supplementary material for: Contribution of major FLM isoforms to temperature-dependent flowering in Arabidopsis thaliana
Source: J Exp Bot. 2017 Sep 25;68(18):5117–27. doi: 10.1093/jxb/erx328 (PMC5853260; doi:10.1093/jxb/erx328)
Supplement: Supplementary_Figures_S1_S2_Supplementary_Tables_S1_S7 [file erx328_suppl_supplementary_figures_s1_s2_supplementary_tables_s1_s7.pdf]

Supplementary Data

Contribution of major *FLM* isoforms to temperature-dependent mediated flowering in *Arabidopsis thaliana*

Giovanna Capovilla, Efthymia Symeonidi, Rui Wu, Markus Schmid

Content:

Fig. S1
Fig. S2
Table S1
Table S2
Table S3
Table S4
Table S5
Table S6
Table S7

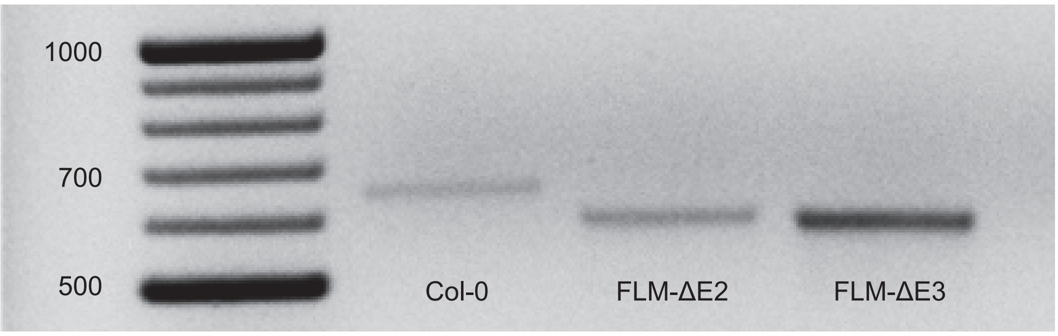

Fig. S1. Detection of CRISPR-Cas9-induced deletions in *FLM*. PCR amplification on the *FLM* genomic region spanning intron 1 to exon 4 shows the 57 bp and 64 bp deletions in *FLM-ΔE2* and *FLM-ΔE3*, respectively.

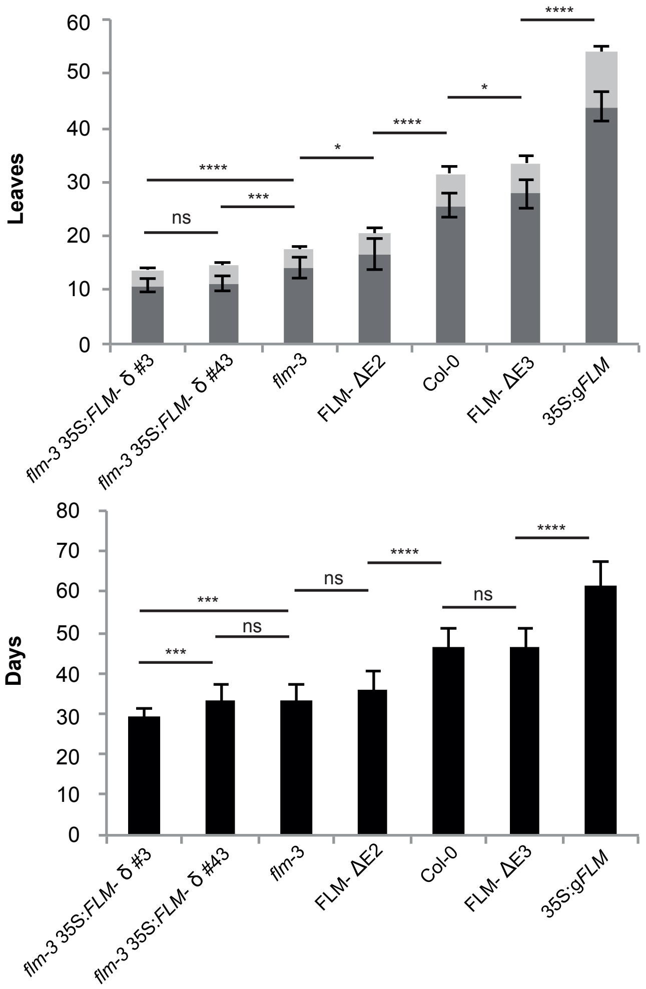

Fig. S2. Flowering time of FLM transgenic lines and mutants at 16°C long days. Flowering time given in number of leaves and days to flowering of the CRISPR/Cas9 lines and the *FLM* over-expressing lines grown at low temperature. Error bars indicate standard deviation. *P < 0.05; **P < 0.01;***P < 0.001; ****P < 0.0001; ns, not significant, using Welch's t-test.

Table S1. List of natural accessions analysed.

Col-0
Oy-0
Can-0
Jl-3
Ra-0
KZ-1
Lag2-2
Van-0
ice73
ice130
ice120
Vie-0
Sei-0
ice70
ice75
ice150
Tsu-0
Bor-4
ice152
Pu2-7
Qui-0
Ey1.5-2
Sha
STAR8
ice71
Tsu-1
Rubezhnoe1
Ru3.1-27
TuSB30-2
ice228
ice1
Ct-1
Mt-0
Nie1-2

Table S2. Oligonucleotides used for TaqMan assay.

Gene	Primer	Sequence (5' to 3')	Fluorophore	Detection (nm)	Quencher
***FLM-β* PROBE**	G-36620	CCAAGATCATTGATCGTTATGAAATACAA	6-FAM™	515-530	BHQ-1
***FLM-δ* PROBE**	G-36229	CGGAGAAACCTCAATGTTTTGAACTC	HEX™2	560-580	BHQ-1
***FLM* Forward**	G-36231	CGCTGTTGTCGTCGTATCTG			
***FLM* Reverse**	G-36743	CTAGTAACTCCTTGTGTGGAAG			
**UBC21 PROBE**	G-38938	GGAGTCCTGCTTGGACGCTTCAGTCTG	CY5®	675-690	BHQ-2
**UBC21 Forward**	G-38780	CTCCTCAAGTTCGATTCTTG			
**UBC21 Reverse**	G-38783	CCTGAGTCGCAGTTAAGAGG			

Table S3. Oligonucleotides used in this work.

Oligonucleotides used for cloning sgRNA constructs:

sgRNA	Oligo	Sequence (5’ -> 3’)
FLM-E2-5’	G-40172	gTCAATGATCTTGGAAATGCgttttagagctatgctg
	G-40173	GCATTTCCAAGATCATTGAcaatcactacttcgactc
FLM-E2-3’	G-40174	gCTTAGCTAATTACTTACTAgttttagagctatgctg
	G-40175	TAGTAAGTAATTAGCTAAGcaatcactacttcgactc
FLM-E3-5’	G-40176	gGCGGTTTTTGGTGTTATGAgttttagagctatgctg
	G-40177	TCATAACACCAAAAACCGCcaatcactacttcgactc
FLM-E3-3’	G-40178	gAACTCTAGAGAATTAAGTTgttttagagctatgctg
	G-40179	AACTTAATTCTCTAGAGTTcaatcactacttcgactc

Oligonucleotides for PCR to detect *FLM* deletions:

Position	Oligo	Sequence (5’ -> 3’)
Intron 1	G-39775	GCACCAGATGATCAGAGTTTCA
Exon 4	G-28145	GATAATTCTGAATTTTTTCTTCAAGATC

Line	PCR product size
Col-0	652
FLM-ΔE2	595
FLM-ΔE3	588

Oligonucleotides for PCR to screen for *FLM* isoforms:

Position	Oligo	Sequence (5’ -> 3’)
Exon 1	G-36231	CGCTGTTGTCGTCGTATCTG
Exon 9	G-28156	CAGCAACGTATTCTTTCCCAT

Oligonucleotides for qPCR to quantify specific *FLM* isoforms:

Position	Oligo	Sequence (5’ -> 3’)
Exon 1 to Exon 4	G-43257	CTTAGAGCCTTAGATCTTGAAG
Exon 5 to Exon 4	G-43258	CTTCAAGCTTGCTTTGGACTG
Exon 1 to Exon 3	G-43259	CCTCCGGTGACGAGATAGAAG
Exon 4 to Exon 3	G-43260	GAATTTTTTCTTCAAGATCGAG
Exon 3 to Exon 4	G-43261	GTTTTGAACTCGATCTTGAAG
Intron 4	G-2641	GAGGGGAGAAAAATGTGTCG

Table S4. Percentage of *FLM* isoforms analysed in Col-0 and CRISPR lines at 16°C, 23°C and 27°C.

Isoform	Col-0 16°C	Col-0 23°C	Col-0 27°C	FLM-ΔE2 16°C	FLM-ΔE2 23°C	FLM-ΔE2 27°C	FLM-ΔE3 16°C	FLM-ΔE3 23°C	FLM-ΔE3 27°C
FLM-β	41.5	48.8	17.2	-	-	-	74.4	64.1	35.4
FLM-δ	26.8	22.0	25.9	70.6	72.7	66.0	-	-	-
ASF1	2.4	-	-	-	-	-	-	-	-
ASF2	2.4	-	-	2.9	2.3	-	-	-	-
ASF3	4.9	-	-	-	-	-	-	-	-
ASF4	4.9	9.8	5.2	-	-	-	-	-	-
ASF5	4.9	2.4	10.3	-	-	-	-	-	-
ASF6	2.4	2.4	-	-	-	-	-	-	-
ASF7	4.9	4.9	8.6	-	-	-	7.7	15.4	25.0
ASF8	2.4	-	-	-	-	-	-	-	-
ASF9	2.4	-	1.7	-	-	-	-	-	-
ASF10	-	4.9	-	11.8	11.4	12.8	-	-	-
ASF11	-	2.4	-	-	-	-	-	2.6	10.4
ASF12	-	2.4	-	-	-	-	-	-	-
ASF13	-	-	3.4	-	-	-	-	-	-
ASF14	-	-	1.7	-	-	-	-	-	-
ASF15	-	-	1.7	-	-	-	-	-	-
ASF16	-	-	1.7	-	-	-	-	-	-
ASF17	-	-	1.7	-	-	-	-	2.6	2.1
ASF18	-	-	1.7	-	-	-	-	-	-
ASF19	-	-	1.7	-	-	-	-	-	-
ASF20	-	-	1.7	-	-	-	-	-	-
ASF21	-	-	1.7	-	-	-	-	-	-
ASF22	-	-	1.7	-	-	-	-	-	-
ASF23	-	-	5.2	-	-	4.3	-	-	-
ASF24	-	-	1.7	-	-	-	-	-	-
ASF25	-	-	1.7	-	-	-	2.6	-	-
ASF26	-	-	1.7	-	-	-	-	-	2.1
ASF27	-	-	1.7	-	-	-	-	-	-
ASF28	-	-	-	2.9	-	-	10.3	2.6	8.3
ASF29	-	-	-	2.9	-	-	-	-	-
ASF30	-	-	-	-	-	2.1	-	-	-
ASF31	-	-	-	-	-	-	-	-	2.1
cASF1	-	-	-	2.9	-	-	-	-	-
cASF2	-	-	-	2.9	-	-	-	-	-
cASF3	-	-	-	2.9	-	-	-	-	-
cASF4	-	-	-	-	4.5	-	-	-	-
cASF5	-	-	-	-	4.5	-	-	-	-
cASF6	-	-	-	-	2.3	2.1	-	-	-
cASF7	-	-	-	-	2.3	-	-	-	-
cASF8	-	-	-	-	-	4.3	-	-	-
cASF9	-	-	-	-	-	2.1	-	-	-
cASF10	-	-	-	-	-	2.1	-	-	-
cASF11	-	-	-	-	-	2.1	-	-	-
cASF12	-	-	-	-	-	2.1	-	-	-
cASF13	-	-	-	-	-	-	5.1	5.1	6.3
cASF14	-	-	-	-	-	-	-	5.1	-
cASF15	-	-	-	-	-	-	-	2.6	2.1
cASF16	-	-	-	-	-	-	-	-	2.1
cASF17	-	-	-	-	-	-	-	-	2.1
cASF18	-	-	-	-	-	-	-	-	2.1
% Tot non canonical isoforms	31.7	29.3	56.9	29.4	27.3	34.0	25.6	35.9	64.6
Number of colonies	41	41	58	34	44	47	39	39	48

Table S5. Flowering time of CRISPR lines.

16°C	Leaves	Cauline leaves	Days	n
flm-3	15.1 ± 1.5	4.2 ± 0.4	36.3 ± 2.3	15
**FLM-ΔE2**	15.8 ± 1.7	3.8 ± 1.1	37.5 ± 3.4	14
**Col-0**	26.9 ± 2.1	5.4 ± 1.2	49.3 ± 3.7	14
**FLM-ΔE3**	33.2 ± 5.2	6 ± 1.6	54.6± 7.3	13
**35S:gFLM**	53.4 ± 3.3	10.4 ± 1.7	62.5 ± 4.6	8
**23°C**	**Leaves**	**Cauline leaves**	**Days**	**n**
flm-3	8.6 ± 1	2.7 ± 0.5	18.8 ± 0.6	16
**FLM-ΔE2**	9.5 ± 1	2.9 ± 0.3	18.9 ± 0.5	16
**Col-0**	13.1 ± 1.3	3.2 ± 0.7	25.1 ± 2.2	15
**FLM-ΔE3**	16.4 ± 2.2	3.6 ± 0.6	27.3 ± 1.6	16
**35S:gFLM**	25.2 ± 2.3	4.9 ± 0.7	35.2 ± 1.5	11
**27°C**	**Leaves**	**Cauline leaves**	**Days**	**n**
flm-3	6.4 ± 0.6	2.4 ± 0.5	14.6 ± 1.4	16
**FLM-ΔE2**	7.4 ± 1	2.8 ± 0.4	15.3 ± 1.5	16
**Col-0**	7.8 ± 1	2.8 ± 0.4	17.8 ± 1.3	16
**FLM-ΔE3**	9.7 ± 0.9	3.1 ± 0.3	20.3 ± 2.6	16
**35S:gFLM**	11.3 ± 1.1	2.6 ± 0.5	23.3 ± 2.1	12

n = number of plants analysed per genotype.

Table S6. Flowering time of natural accessions

Accession	TL16	D16	TL23	D23	TL27	D27	n16	n23	n27
Col-0	23.2 ± 2.7	35.3 ± 3.2	13.4 ± 1.5	19.2 ± 1.6	10.4 ± 1.2	15.7 ± 1.8	30	30	26
Can-0	45.9 ± 3.7	52.6 ± 7.4	26.1 ± 6.7	29.6 ± 2.6	14.1 ± 1.8	18.7 ± 3.6	13	15	15
Ra-0	40.8 ± 5.3	50.5 ± 6.6	12.5 ± 2.1	19.3 ± 4	10.3 ± 1	17.8 ± 2.8	4	4	4
KZ-1	22.8 ± 3.8	64.5 ± 9.3	15.3 ± 2.6	22 ± 2.4	12.8 ± 2.8	26 ± 8.8	4	4	4
Lag2-2	41 ± 14	72 ± 13	14.3 ± 1.3	22.8 ± 2.9	10.3 ± 0.5	17.3 ± 2.2	4	4	4
Van-0	20.3 ± 1.7	33.6 ± 2.5	16.8 ± 3	20.7 ± 1.5	9.7 ± 1.4	17.8 ± 3.7	25	29	27
ice73	50.3 ± 3.5	67 ± 2.6	35.5 ± 9.7	41.8 ± 8.4	29 ± 8	40.8 ± 8.6	3	4	4
ice130	50.3 ± 5.4	71 ± 3.7	41.3 ± 3.1	63.3 ± 13.6	34.5 ± 4.9	47.5 ± 7.9	4	3	4
ice120	64.5 ± 2.4	69.8 ± 1.9	60.3 ± 9.3	69.5 ± 6.9	53.7 ± 8.1	57 ± 15.6	4	4	3
Vie-0	76.5 ± 1.3	77.8 ± 8.1	59.7 ± 4.6	65.3 ± 12.9	52.5 ± 6.2	78.3 ± 6.5	4	3	4
Oy-0	53.3 ± 7.3	51.7 ± 5.2	20.5 ± 3.2	21.3 ± 1.7	14 ± 1.1	18.6 ± 0.7	29	27	29
Sei-0	16.9 ± 2.6	32.3 ± 2.2	10.6 ± 1.7	16.3 ± 1.4	9.4 ± 1.1	14.6 ± 3.8	27	30	28
ice70	56.5 ± 15.4	78.8 ± 11.3	55.3 ± 7.2	60.3 ± 4.2	44.3 ± 5.5	52 ± 8.5	4	3	4
ice75	53 ± 4.4	70 ± 6.1	52.5 ± 8.7	58.8 ± 6.9	31 ± 6.4	40.8 ± 8.5	3	4	4
ice150	51 ± 3.6	65.8 ± 2.5	45 ± 10.9	58.3 ± 9.4	41.3 ± 4.9	59 ± 6.7	4	4	4
Tsu-0	53.5 ± 7.8	65.5 ± 7.6	23.8 ± 4.1	26.3 ± 2.2	16.3 ± 1.5	21.5 ± 1.3	4	4	4
Bor-4	63.5 ± 4.7	65 ± 5.4	36 ± 2.2	34.3 ± 1.7	20.5 ± 2.4	21.5 ± 1.3	4	4	4
ice152	50.3 ± 4.9	65.5 ± 4	41.3 ± 3.5	69.3 ± 14.2	33.3 ± 4.5	47.7 ± 6	4	4	3
Pu2-7	57.5 ± 9.2	77.5 ± 7.8	42.5 ± 1.7	45.3 ± 6.8	38.3 ± 3.2	47 ± 4	3	3	3
Qui-0	62.8 ± 10.1	61.5 ± 9.8	45.3 ± 17.8	46.3 ± 16.6	34.3 ± 24.2	36 ± 23.6	4	3	4
Ey1.5-2	50.7 ± 3.1	71.8 ± 10.7	16.5 ± 1.7	23.5 ± 1.9	14 ± 0.8	20 ± 2.2	3	4	4
Sha	19.8 ± 3	44.8 ± 3.2	9.5 ± 0.6	24.5 ± 1.3	8.5 ± 1	19.5 ± 1.7	4	4	4
STAR8	38.5 ± 8.4	49.5 ± 2.6	22 ± 4.7	30.3 ± 5.9	27.3 ± 7.9	36.5 ± 9	4	4	4
ice71	46 ± 10.7	68 ± 12.2	42.8 ± 5.3	47.5 ± 5.7	32.3 ± 3.1	45.3 ± 4.3	4	4	4
Tsu-1	53.3 ± 5.4	66.3 ± 11.6	20.8 ± 2.1	25.5 ± 1.9	14.3 ± 1.3	21.3 ± 2.5	4	4	4
Rubezhnoe-1	51 ± 3.2	54.3 ± 3.3	34 ± 12	35.3 ± 8.5	22.8 ± 3.2	28.5 ± 8	4	4	4
Ru3.1-27	42.8 ± 4.1	51 ± 4.8	21 ± 1.8	22 ± 2	15.3 ± 3.4	21 ± 4.1	4	4	4
TuSB30-2	39 ± 2	52.3 ± 2.1	17.8 ± 2.6	25 ± 1.4	12 ± 1.8	21.5 ± 2.4	3	4	4
ice228	62.8 ± 6.5	60.8 ± 4.8	55 ± 2	61.7 ± 7.6	48.5 ± 4.9	96.5 ± 13.4	3	3	3
ice1	64 ± 5.3	64 ± 2	60.7 ± 1.5	69.7 ± 0.6	48 ± 5.7	71 ± 2.8	3	3	4
Ct-1	37 ± 3.4	64 ± 4.8	11.8 ± 1.5	21 ± 2.7	10.8 ± 1	20 ± 0.8	4	4	4
Mt-0	21.8 ± 2.9	40.8 ± 3.6	12 ± 1.4	18.5 ± 1.3	11.3 ± 1.3	17.5 ± 3	4	4	4
Nie1-2	34 ± 8.8	61.3 ± 9.1	15.3 ± 4	22.8 ± 6.3	11.8 ± 2.9	27.8 ± 6.7	4	4	4

TL16, TL23 and TL27, total leaf number at 16°C, 23°C and 27°C; D16, D23 and D27 days to flowering at 16°C, 23°C and 27°C; n, total number of plants analysed per accession per temperature.

Table S7. Flowering time data of FLM transgenic lines and mutants at 16°C long days.

	total leaves	rosette leaves	cauline leaves	days	TPN
flm-3	17.4 ± 2.5	14.1 ± 1.9	3.3 ± 0.8	33.4 ± 3.5	17
FLM-ΔE2	20.4 ± 3.9	16.6 ± 2.8	3.8 ± 1.2	35.7 ± 4.8	18
Col-0	31.6 ± 2.3	25.4 ± 2.2	6.1 ± 1.1	46.5 ± 4.4	16
FLM-ΔE3	33.5 ± 3.4	27.7 ± 2.6	5.8 ± 1.2	46.7 ± 4.2	15
35S:g*FLM*	53.9 ± 2.6	43.8 ± 2.7	10.1 ± 1.2	61.5 ± 5.8	18
flm-3 35S:*FLM-δ* #3	13.7 ± 1.8	10.8 ± 1.4	2.9 ± 0.6	29.0 ± 2.2	25
flm-3 35S:*FLM-δ* #43	14.4 ± 1.7	11.2 ± 1.4	3.2 ± 0.7	33.3 ± 3.7	15
